# Using Natural Language Processing to Identify Symptomatic Adverse Events in Pediatric Oncology: Tutorial for Clinician Researchers

**DOI:** 10.2196/70751

**Published:** 2025-07-24

**Authors:** Clifton P Thornton, Maryam Daniali, Lei Wang, Spandana Makeneni, Allison Barz Leahy

**Affiliations:** 1 Center for Pediatric Nursing Research & Evidence-Based Practice, Children's Hospital of Philadelphia, 734 Schuylkill Avenue, Philadelphia, PA, 19104, United States, 1 2674266188; 2 School of Nursing, University of Pennsylvania, Philadelphia, PA, United States; 3 Department of Biomedical and Health Informatics, Children's Hospital of Philadelphia, Philadelphia, PA, United States; 4 College of Computing and Informatics, Drexel University, Philadelphia, PA, United States; 5 Perelman School of Medicine, University of Pennsylvania, Philadelphia, PA, United States; 6 Cancer Center, Children's Hospital of Philadelphia, Philadelphia, PA, United States

**Keywords:** neoplasms, artificial intelligence, natural language processing, interdisciplinary research, oncology

## Abstract

Artificial intelligence (AI) is poised to become an integral component in health care research and delivery, promising to address complex challenges with unprecedented efficiency and precision. However, many clinicians lack training and experience with AI, and for those who wish to incorporate AI into research and practice, the path forward remains unclear. Technical barriers, institutional constraints, and lack of familiarity with computer and data science frequently stall progress. In this tutorial, we present a transparent account of our experiences as a newly established interdisciplinary team of clinical oncology researchers and data scientists working to develop a natural language processing model to identify symptomatic adverse events during pediatric cancer therapy. We outline the key steps for clinicians to consider as they explore the utility of AI in their inquiry and practice, including building a digital laboratory, curating a large clinical dataset, and developing early-stage AI models. We emphasize the invaluable role of institutional support, including financial and logistical resources, and dedicated and innovative computer and data scientists as equal partners in the research team. Our account highlights both facilitators and barriers encountered spanning financial support, learning curves inherent with interdisciplinary collaboration, and constraints of time and personnel. Through this narrative tutorial, we intend to demystify the process of AI research and equip clinicians with actionable steps to initiate new ventures in oncology research. As AI continues to reshape the research and practice landscapes, sharing insights from past successes and challenges will be essential to informing a clear path forward.

## Introduction

The development of sophisticated machine learning, deep learning, natural language processing (NLP), and large language models has showcased artificial intelligence’s (AI’s) potential to accelerate advances in health care research and clinical practice [1-3]. However, growing clinician interest in employing AI as a research tool is often met with challenges in understanding its nuances and applications. The proper and safe use of AI requires in-depth knowledge of computer science, big data analytics, and specialized data science and biostatistical approaches – skills that clinicians typically do not possess. Conversely, computer and data scientists with expertise in AI who wish to contribute to clinical advances must develop familiarity with a clinical specialty and acquire a deep understanding of the intricacies of care delivery, research, and biomedical needs. As a result, the effective use of AI in health care environments necessitates collaborative integration between computer science and health care disciplines, bringing together expertise from these disparate fields [4-6].

Although clinicians are increasingly eager to incorporate AI into their research efforts, many face uncertainty on how to begin or establish effective collaborations with computer and data scientists. Using the initial phase of our pilot AI work as an exemplar, we outline strategies for leveraging AI and NLP in pediatric cancer inquiry, focusing on the process of building a team blending AI and clinical oncology research. Our transparent account details the formation of an interdisciplinary team bridging clinical oncology and data science, highlights challenges encountered, and shares lessons learned. The purpose of this descriptive tutorial is to make AI approachable for clinical researchers who are motivated to address complex clinical questions but may lack technical expertise. Key challenges for teams to consider are explicitly identified within this study. We aim to equip clinician readers with an introductory framework for initiating AI-driven research projects, while emphasizing the logistic, financial, and personnel resources essential for success.

## The Clinical Problem and Need for an AI-Based Solution

Cancer-directed therapy is inherently toxic, causing a host of adverse events that are burdensome, costly, dangerous, and sometimes life-threatening [7-9]. When toxicities become severe, future therapy doses are reduced, delayed, or omitted, which potentially compromises long-term survival [10]. Because of these deleterious effects, research focused on early detection has been prioritized, so that prompt and effective interventions can be designed to mitigate toxicity and improve clinical outcomes [11-13].

Therapy-related toxicities are broadly categorized into nonsymptomatic and symptomatic adverse events. Nonsymptomatic adverse events are objective and easy to identify, quantify, and analyze because they are readily detectable through structured data like laboratory values or diagnostic imaging. These clean and structured data allow researchers to stratify patient cohorts, correlate symptoms with biomarkers and treatment factors, and derive actionable insights.

In contrast, symptomatic adverse events are subjective and must be elicited, interpreted, or individually assessed by clinicians [14,15]. Furthermore, these events are typically captured in unstructured, free-text clinical notes which constrains systematic identification and analysis, making data extraction labor-intensive, time-consuming, and prone to inconsistencies [7-9,16,17]. Not surprisingly, the data are often unreliable [18,19], with significant negative repercussions on subsequent analyses. The inability to reliably study symptomatic adverse events is particularly concerning because they are among the most common therapy-related toxicities and frequently lead to treatment interruptions.

AI is a promising method for the reliable extraction and analysis of symptomatic adverse events from electronic medical records (EMRs). In fact, NLP technology has already had preliminary success in identifying their presence within unstructured, free-text clinical notes [20-24].

In pediatric oncology, 5 symptomatic adverse events associated with chemotherapy stand out due to their prevalence and serious sequelae—nausea, vomiting, constipation, neuropathy, and mucositis. Herein, we describe our interdisciplinary approach for assessing the ability of an NLP algorithm to identify these adverse events in pediatric oncology patient records. The initial phase of this work, serving as the exemplar for this tutorial, is to evaluate the degree to which existing NLP models can identify symptomatic adverse events in pediatric cancer therapy.

## Infrastructure, Personnel, and Funding

AI-based health care research necessitates substantial data and computer science support. Optimally, this support is institutional, with health care enterprises investing in employing, contracting, or collaborating with skilled data scientists dedicated to advancing clinical inquiry. Collaboration between these technology experts and clinician researchers, along with departmental backing to support clinical inquiry and innovation, as well as the necessary data infrastructure, is essential to cultivating advancements in this emerging domain [25].

Our institution houses a Data Science and Biostatistics Unit (DSBU), a centralized service unit that comprises a robust mix of 30 PhD- and master-level biostatisticians and data scientists who work with principal investigators to address research questions via data consultation, study design, methodology expertise, data preparation, data analyses, and manuscripts development. The DSBU is housed within the Department of Biomedical and Health Informatics, which provides an academic home and service base for all research informatics activities at the institution, including the development and deployment of intellectual, technical, and educational resources in biomedical computing.

Through an enterprise-level strategic initiative, our institute developed a next-generation suite of tools and services, Arcus, that provides a digital laboratory environment for investigators and project staff to securely store, access, and process electronic patient data. The Arcus program is staffed by archivists, librarians, information analysts, cloud computing engineers, programmers, statisticians, and privacy experts. Data are managed through the oversight of the Institutional Review Board (IRB), and access is governed by multiple institutional policies. Arcus security configuration and controls are based on the HIPAA (Health Insurance Portability and Accountability Act) Security Rule.

For this work, project team members from DSBU and Arcus included 3 PhD-prepared data scientists and a data integration manager. Initial services to set up the project were provided at no cost through the internal consultation mechanisms. As the project developed and expanded, pilot funding was secured through internal grant mechanisms and preliminary data were used to secure external grant funding. A data science supervisor available through the center provided guidance in approaching an AI-based research project.

The project team’s clinical experts were 2 oncology clinicians and researchers who served as coprincipal investigators—a PhD-prepared scientist and nurse practitioner under the Center for Pediatric Nursing Research and Evidence-Based Practice and Cancer Center and an attending pediatric oncologist in the Division of Pediatric Oncology and School of Medicine.

## Building the Digital Laboratory

The clinician researchers consulted with the data science team extensively to determine necessary data elements and ensure feasibility. An IRB application was submitted, the research was determined to meet exemption criteria, and a HIPAA waiver was authorized (IRB 24‐021922).

**Figure 1.**
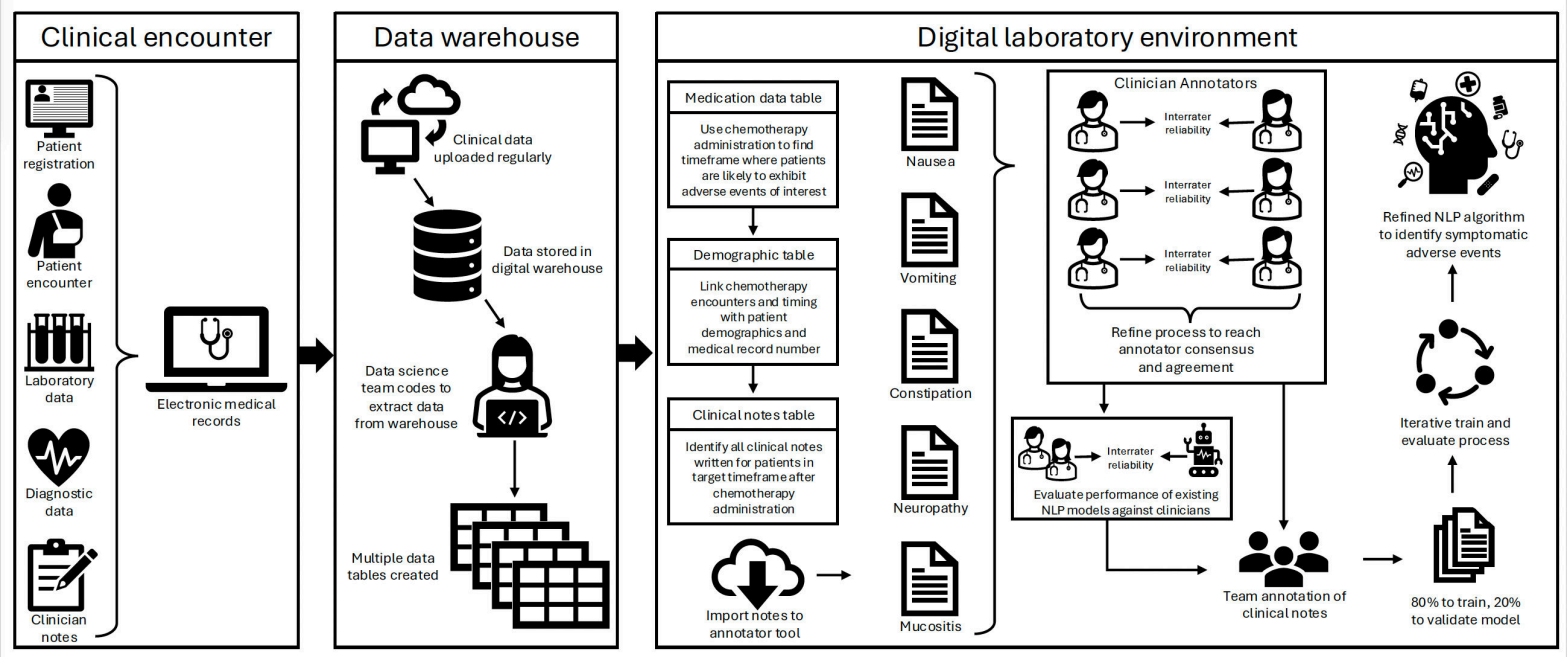
Research project progression and data flow from clinical encounters to the data warehouse, and manipulation within the digital laboratory environment. NLP: natural language processing.

Activities relating to building the digital laboratory, including data flow and processing, are outlined in Figure 1. Inclusion criteria were set to any patient aged younger than 25 years who received treatment for cancer at our institution within the previous 10 years. We used *International Classification of Diseases, Ninth Revision* (*ICD-9*) or *International Classification of Diseases, Tenth Revision* (*ICD-10*) diagnosis codes, Current Procedural Terminology codes for cancer-directed therapies in conjunction with institutional cancer registry data to identify those who received cancer treatment (“Clinical Encounter” in Figure 1). Eligible patients were assigned a unique identifier and added to the digital laboratory. Importantly, each unique identifier retained a link to the patient’s electronic health record (EHR) medical record number to ensure reliable linking of patients with relevant clinical data. Necessary EHR data elements (eg, chemotherapy administration records, clinical notes, and laboratory values) were identified via joint clinical and data science team meetings and were then imported from the data warehouse into the digital environment (“Data Warehouse” in Figure 1).

Although careful planning to meet aims is necessary for all research projects, big data and AI-based research involves the additional step of evaluating the accessibility and reliability of data. A key challenge in building a digital lab is the extensive refinement of data that is required because digital storage of medical data differs from digital data display (the way data appears to the clinician in the EHR). Within the data warehouse, clinical notes are sorted and stored based on their version status as templated, signed, addended, or modified – with each note potentially possessing multiple versions. But in the clinical setting, the only note displayed for staff is the most recent version. Therefore, to ensure data matched the clinical documentation, the data science team wrote complex code that selected the most recent version, irrespective of its assigned status. This was essential because there are millions of source notes for this work and importing multiple versions of each is not feasible due to time, data storage, and computational processing limitations.

Chemotherapy agents were identified using medication classification codes created for the purpose of this work and then integrated with patient medication administration records to identify the specific administration time and dose. This vital step underscores the need for a skilled data scientist or analyst to be an integral member of the research team. Laboratory values, easily extracted from the source EHR data warehouse, also were imported to assist clinical researchers with interpretation of data, as needed.

After 14 months of collaborative effort, all data were imported to the laboratory (“Digital laboratory environment” in Figure 1) which included data on 18,408 patients, encompassing 4.8 million clinical notes and over 450 million medication dose administrations. From this point forward, all research activities were performed in the digital laboratory environment. It should be noted that due to the massive size of EHR data files and the sheer number of individual variables, discrete data elements are imported to the digital laboratory in the form of tables in a relational database. For example, the medication administration table comprised dozens of datapoints for each of the hundreds of millions of doses administered within our patient cohort. Similarly, the demographic information table contained dozens of variables and associated metadata for each patient. The clinical notes table not only included the full note text, but also other metadata that provided information about the notes themselves (eg, timestamps, subtype, and author type).

Identifying the relevant and necessary data elements from these tables and joining them in relational databases required the expertise of a PhD-level data scientist with fluency in programming and querying in SQL and R languages. The clinician scientists provided direction for selecting elements but did not have the skill to perform the tasks. Once relational tables and databases were created, the team could jointly verify data integrity through face validity of items (eg, chemotherapy agents matched oncologic diagnoses for patients). The team also reviewed randomly selected medical records of patients in the database to ensure correct elements and values were identified and joined appropriately in the newly created data tables.

During the process of building the digital laboratory, unexpected challenges arose from the complexity of the structures of EHR data and the differences between digital data storage and display that complicated data pulling and importing approaches. The complexity of identifying and pulling these data was also underestimated by the data science team, and the process took much longer than expected, by a scale of about a year. Clinician researchers taking initial steps to AI-based methods should account for time required to learn new skills and take additional time to clean and validate data. However, the accessibility to data scientist and technology expert knowledge, skills, and time coupled with the infrastructure provided by institutional investments and external grant funding made the project both feasible and possible.

## Identifying Notes of Interest

Training and evaluation of the NLP model is an iterative process requiring labeled data. For this project, the labeled data are annotations of text, wherein a clinician reads through clinical notes and tags sections that indicate the absence, presence, and severity of the adverse event of interest. A typical allocation of 80% of annotated notes for model training and 20% for validation was used. The necessary number of labeled notes varies considerably depending on the complexity of the task (ie, difficulty of being able to identify the adverse event of interest) and the selected NLP methodology. For these reasons, it is not possible to a priori estimate the minimum number of notes required to adequately train and validate the model. Thus, we used an incremental annotation process starting with a minimum sample size for a limited population similar to previous work [26]. For clinician researchers accustomed to a priori–determined sample sizes, this was difficult to conceptualize and resulted in downstream challenges in time management and resource allocation for the project. Adopting a qualitative research mindset – where recruitment is ongoing until data saturation is achieved – is helpful when conceptualizing sample size for a project like this, despite being a technique not used frequently in quantitative methodologies.

**Figure 2.**
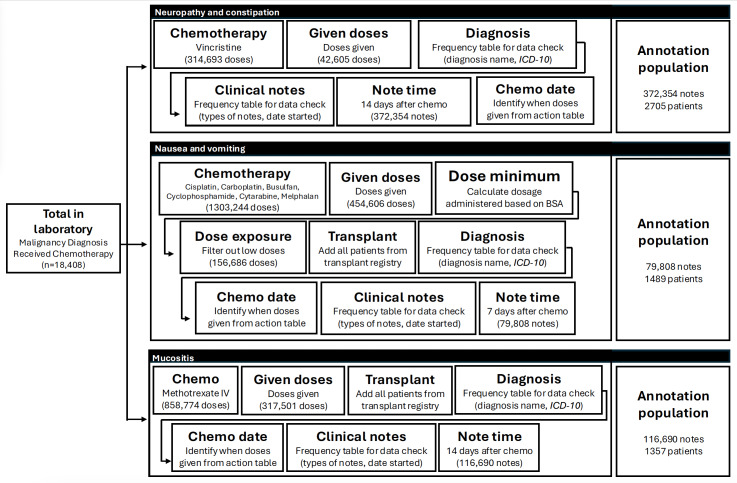
Schema for identifying clinical notes to annotate for natural language processing training. BSA: body surface area; *ICD-10*: *International Classification of Diseases, Tenth Revision.*

The process of identifying notes for annotation required several months and the expertise of a PhD-prepared data scientist skilled in coding and data analysis. Our goal was to identify notes with a high likelihood of containing documentation related to the adverse events of interest to facilitate faster model training. As such, clinical researchers identified key scenarios and exposures associated with nausea, vomiting, constipation, neuropathy, and mucositis. This process involved specifying chemotherapy agents, dosages, and the typical time frames within which these toxicities manifest following administration. The schema used for identifying notes meeting these criteria is outlined in Figure 2.

To identify clinical notes most likely to document constipation or neuropathy, we identified instances of vincristine administration. For nausea and vomiting, highly emetogenic chemotherapy agents were identified [27]. Given that emetogenicity depends on dosage, body surface area was calculated using the most recent height and weight measurements, and doses below the emetogenic threshold were excluded. Patients undergoing conditioning chemotherapy for stem cell transplantation, which is universally highly emetogenic, were included based on an institutional transplant registry. To identify documentation of mucositis, we focused on intravenous methotrexate administrations as well as stem cell transplantation.

Chemotherapy doses were identified from the medication table, and a frequency table of administration events, including action and date and time, was reviewed to ensure proper documentation (eg, marked as “given” in the medical records). Determination of how administered medications are recorded in the data warehouse required consultation with an informaticist, since multiple actions (eg “missed,” “late,” “withheld,” “administered,” and “given”) are assigned to medications in the dataset with ambiguous meanings. Cross-referencing with data visible in the EHR was required to ensure that the devised algorithm and decisions were made. As before, the clinical researchers learned that data stored in the data warehouse is far more complex than that which is displayed in the EHR. Identifying administered medications within the medication administration record in the “visible” EHR, for example, is far more straightforward, but incredibly labor-intensive.

Patient identifiers were cross-referenced with demographic and diagnosis tables, followed by the generation of a frequency table of oncologic diagnoses and associated *ICD-9* and *ICD-10* codes for each target symptom. Clinical researchers reviewed these tables for errors or incongruences to establish face validity, ensuring that chemotherapy agents matched the diagnoses. After validating these data, we cross-referenced the clinical notes table using patient identifiers to extract notes written within 14 days of chemotherapy administration for neuropathy, constipation, and mucositis; and 7 days for nausea or vomiting, in accordance with expected clinical timelines.

Initial review suggested that certain note types—such as history and physicals, progress notes, nursing notes, and discharge summaries—were most likely to contain relevant data. However, inconsistencies in data labeling posed challenges; for instance, “progress notes” were used for documentation by multiple specialties, adding noise to the dataset. After careful review, notes authored by clinical nutritionists, pharmacists, social workers, case managers, speech and language pathologists, occupational therapists, and physical therapists were excluded. Only the most recent version of each note (signed, addended, or modified) as determined by date of note initiation and note status was retained.

Key challenges to identifying relevant note types, versions, and authors arose from the time-intensive nature of extensive data extraction and manual review required. Clinical staff encountered challenges in understanding how medical record data were stored within the data warehouse, particularly regarding labeling of note versions and determining when patients received medications. Overcoming this challenge highlights the importance of properly understanding the metadata that accompanies variables of interest, and the parallel importance of including all metadata in the digital laboratory. As before, the team learned that the vocabulary typically used in the clinical environment does not match that used in informatics. For example, in clinical practice, “administered” or “given” are used synonymously to indicate that a patient has received a medication. However, these had different meanings in the data warehouse, so understanding how data are labeled and not making assumptions is vital. Validating the data by reviewing constructed tables and comparing them to patient medical records is necessary to ensure the integrity of the data. These are both nuanced and time-consuming steps that should be considered as expected components of all big data or AI-based research projects.

Real-time collaboration with a dedicated data scientist enabled efficient extraction and validation of large datasets. The integration of this expertise allowed for immediate adjustments based on clinical input, ensuring that the final dataset was both comprehensive and focused and underscored the importance of interdisciplinary collaboration and iterative problem-solving.

## Annotation and Validation

An annotation guide was created by the clinician researchers to standardize the annotation process and ensure consistency in identifying and grading adverse events. The guide aimed to provide clear instructions for clinical abstractors and facilitate uniform application of the National Cancer Institute’s Common Terminology Criteria for Adverse Events (CTCAE) [28] to patient records.

The guide was created iteratively, beginning with an initial draft used by clinician researchers during joint annotation sessions. Common challenges encountered during annotation were documented, and adjudication decisions were included to ensure consistency. Common data extraction elements that required discussion among clinicians were included in the guide to define consensus between researchers and to provide consistency to annotators. The guide accounts for nuances of clinical documentation such as shorthand abbreviations, terminology variations, and physical exam findings. To initiate the annotation process, 100 notes, representing an intersection of chemotherapy exposures associated with all the target adverse events, were uploaded to the annotation tool. Clinicians independently annotated 30 notes, comparing results to assess alignment that facilitated refinement of the annotation guide before independently completing the remaining 70 notes. Annotation overlap and agreement were systematically evaluated, with areas of disagreement manually adjudicated and further revisions made to the guide.

A second and third batch of 100 notes was then annotated independently and annotator agreement calculated after each round. Annotator agreement was evaluated by interrater reliability calculated by tag agreement at the symptom level (constipation, mucositis, nausea, neuropathy, and vomiting) and at the symptom degree level (eg, CTCAE severity level). Weighted Cohen kappa quantified the level of agreement to provide a measure of agreement accounting for the likelihood of agreement occurring by chance. Manual adjudication after each round was then undertaken, followed by revision of the annotation guide. Discrepancies were explored to identify opportunities for improvement and additional nuances in clinical documentation.

Unexpectedly, initial low agreement between abstractors highlighted challenges in applying CTCAE criteria to retrospective medical records. This partially stemmed from the format of notes in the annotator tool. Because they were removed from the EMR system, there was an inability to incorporate contextual data typically used by clinicians to make severity assessments. Administration of as-needed medication, for example, was not always apparent in free-text clinical notes. Such ambiguities are inherent to retrospective reviews and reflect broader limitations in applying clinical grading systems to medical record data, but the iterative approach facilitated the creation of a detailed annotation guide and established a reliable methodology for future annotation efforts. The complexity of these clinical scenarios underscores the need for expert clinicians to remain closely involved with annotations when training AI models.

This study used a modified version of an open-source NLP pipeline, clinical text analysis and knowledge extraction system (cTAKES) [29], as a baseline for comparison against clinician annotations and our novel AI-based model in phenotyping constipation, mucositis, nausea, neuropathy, and vomiting. While cTAKES offers a valuable NLP solution for clinical text, its default configuration is computationally intensive and unsuitable for large-scale datasets. Our existing pipeline addressed this limitation by implementing a distributed processing pipeline capable of handling millions of clinical notes. It also further enhanced cTAKES by incorporating the human phenotype ontology to improve entity recognition and improving the negation annotator to refine accuracy in identifying negated findings [30,31]. This modified cTAKES pipeline served as a baseline for evaluating the performance of our novel transformer models.

With the revised annotation guide and further adjudication between annotators, *F*_1_-scores could be assessed between our baseline NLP model and the clinician annotators. The *F*_1_-score accounts for both sensitivity and recall of an NLP model. The existing off-the-shelf NLP model (cTAKES) was unable to reliably identify symptomatic adverse events of interest for pediatric oncology patients based on interrater reliability, Cohen kappa, and *F*_1_-score analyses. This is clinically problematic, as reliable identification would be necessary for clinical work and to use this model for research purposes. Furthermore, the model is unable to identify symptom severity, further highlighting a need for the development of a fit-for-purpose novel NLP model which is proposed as stage 2 of this study.

## Barriers and Lessons Learned

The first phase of this work provides valuable findings that justify continued research in this area. Our experiences as a newly developed transdisciplinary research team offer insights relevant to other teams that are beginning to integrate AI technologies into clinical research. Table 1 provides a review of our key challenges and the associated implications specific to this work.

**Table 1.** Key challenges, impact specific to this project, and facilitators for success in overcoming challenges.

Key challenge and implications	Facilitator
**Require substantial data and computer science support**	
Clinician scientists and researchers with limited knowledge in computer science and big data methodology	In-house Data Science and Biostatistical Unit with PhD- and master-level biostatisticians and data scientists
Cost associated with collaborative efforts and time of external experts	Free data science consultation for clinical investigators and internal pilot funding that allowed securement of external grants
Platform to manage very large data files and analyze millions of datapoints in analyses	Enterprise-level strategic initiative developed a suite of tools and services for large-scale data analyses
**Complexity of data structures between electronic health records and data warehouse**	
Multiple versions of millions of clinical notes needed to be reviewed to select the correct version	Collaborative effort between PhD-prepared data scientist who coded and executed the tasks and clinicians who validated the output
Chemotherapy agents need to be identified and incorporated to patient selection as part of inclusion criteria	Senior data integration analysts created bespoke labeling system to identify all chemotherapy agents
Clinical data and associated metadata are stored in massive, discrete data tables	PhD-level data scientists with skills in variable identification, database management, and creation of relational databases
Extensive time for database creation and importing of large files to create a workable data model	Flexible timelines and expectations, mutual goals and understanding, and a data model that supports ongoing addition of new data elements
**Inconsistent or misunderstood data labeling in the warehouse**	
Validate research data to ensure consistency with clinical entry formats	Data extraction from the data warehouse and then validated against medical records by clinician staff
Extensive filtering of data elements to ensure integrity of data used for research purposes	Real-time collaboration between data scientists and clinician team members to refine and validate data filtering
**Subjective nature of clinical interpretation of patient scenarios**	
Lack of contextual data available for clinical symptom evaluation	Expert clinicians are required to annotate text for model training
Consistent method is needed to identify outcomes of interest to train AI^a^ models	Creation of an annotation guide and consistent ontology
Multiple targets for annotation, creating a complicated validation process	Annotation tools and software as standard components of the digital lab environment
Transparent assessment of agreement for decision making between clinicians	Annotation review by expert clinicians to assess performance before model training and evaluation
**Bridging distinct scientific domains to enable unified project execution**	
Mutual understanding of priorities, feasibility, and methodology between data science and clinical research team members	Open, clear, and respectful communication; time to understand terminology and needs; flexible timelines and ongoing dedication from all research team members

a AI: artificial intelligence.

Barriers that slowed progress were primarily related to the inevitable learning curves encountered when embarking on a novel line of inquiry or acquiring a new skill set. The clinical researchers underestimated the time required to develop proficiency in these new methods and the time-intensive nature of interdisciplinary communication. Considerable effort was needed to understand how raw data are stored, transformed, and imported into a digital laboratory. This is noteworthy, not just for planning purposes for other teams, but also in understanding that data labeling and storage is unique to both the individual EMR platforms and the health institutions that use them. This makes the algorithm we have developed for identifying clinical notes specific to our institution and not likely directly transferrable to other sites. However, our methodology and approach can be replicated using institution-specific data elements and metadata, but this will require ongoing time investment.

Key challenges relating to the building of the digital laboratory related to the need for complex coding to identify appropriate clinical notes, the development of novel codes to identify chemotherapy agents, extensive data cleaning and refinement, and time-intensive data validation activities. Variations between how data are presented in the live, front-end version of EMR systems and how they are transformed and stored in the data warehouse created difficulty in translating between these views and ensuring the data accessed were accurate and correct. Logistic challenges related to data acquisition and organization arose from the size of datasets and tables because they included vast amounts of metadata in their raw form and extensive time for the data team to identify appropriate sources for importing. These challenges were overcome by continual partnership between clinical and data science team members and ensuring mutual understanding of needs before each phase of work. Unfortunately, these unanticipated difficulties extended the project’s timeline beyond what was initially anticipated.

Similarly, substantial time was dedicated to ensuring that the data science team comprehended the clinical scenarios underpinning this work. This reflexive exchange was critical for troubleshooting, planning data extraction, and conducting validation activities for model training. As a result, establishing the digital laboratory took significantly longer than anticipated, requiring adjustments to project timelines. Working meetings often focused on aligning terminology and achieving a mutual understanding of project milestones, underscoring the importance of interdisciplinary fluency. Finally, as with many research projects, cost considerations posed challenges. Incentives for clinicians to annotate notes could facilitate a larger group of trained annotators or dedicated research assistants, accelerating the process of achieving an adequate sample size for model training.

As AI becomes increasingly embedded in clinical practice, these models may become core components of clinical and research training programs, underscoring the need for ongoing interdisciplinary collaboration between data scientists and clinicians. These advancements signal an exciting future for AI-driven methodologies in improving patient care and advancing clinical research.

## Facilitators and Necessary Infrastructure

Key facilitators to successfully completing the initial phase of our pilot work are also summarized in Table 1, matched to the implications of this project. They mainly included robust data science infrastructure and support in addition to flexibility of time and working toward mutual understandings. The DSBU and Arcus teams supplied critical expertise, technology, and financial resources, which were leveraged to scaffold this research project and are noted essential components of this type of collaborative work [25]. The clinical researchers defined a research question amenable to AI solutions, fostering a synergistic collaboration between the teams. A balance of funding and accessible resources is needed such that a researcher can either have access to the data science personnel or be able to contract with them for research purposes. These resources enabled our team to establish relationships, evaluate feasibility, and begin data harvesting to generate preliminary data that ultimately secured external funding. Once established, ongoing collaboration, shared priorities, and mutual commitment among team members facilitated a unified direction forward and long-term engagement in the project.

Clinician investigators who desire to engage with AI research need to have affiliation with an organization that has embraced and built an environment to support this work. Doing so requires the organization to make significant financial and personnel investments and overcome several hurdles and barriers to build a team that can orchestrate a large AI platform. Organizations must first determine that the clinical or financial benefits from an AI platform outweigh the upfront costs and long-term risks, requiring a long-term investment mindset [32]. Typical approaches involve identifying AI as a potential useful tool for improving the execution of daily operations and, once instituted, can be used as a research platform. It is therefore primarily integrated to an organization as part of reengineering business processes [33], although there are cases of initiating AI platforms for research purposes as a primary objective. In either case, primary concerns and challenges are typically related to cost, confidentiality and security, data integration and system compatibility, and trustworthiness.

Upfront costs for AI infrastructure are high. Computational resources and power for initial training of algorithms are much higher than later simple execution of the models [34]. Lengthy time to production or to see benefit can deincentivize companies from investments [32], especially when considering that benefits and success are subject to time and other costly factors like computational power [35]. Computational resources, staff, personnel, training, and ongoing maintenance – including data audits, revised learning algorithms, ongoing data management, and updates – further add cost to AI adoption across a multitude of industries [32,34,35]. For these reasons, some smaller pharmaceutical companies, for example, have declined integration because the upfront costs are too high, unlike their larger counterparts, who see significant financial gain from even a small amount of process improvement [33]. However, taking strategic recommendations from end users, ensuring that there are well-defined problems amenable to AI-based solutions, and ensuring clear objectives for its use ensure valued return on investment [32,36].

Beyond cost, confidentiality and data security are of paramount concern, especially in health systems that are subject to stringent privacy laws and ethical considerations [6,34-36]. Safeguarding patient information requires legal counsel, information security personnel, and computer scientists. Similarly, these resources assist with concerns of data integration and system compatibility, ensuring that the AI platform can accept, synthesize, and augment existent data and work synergistically with programs already in use. For health care, this includes the EHR system, radiology software, mobile apps, pharmacy programs, billing systems, and scheduling programs.

Finally, uptake and integration of AI are halted if there is concern about the trustworthiness of the programs or if users – inclusive of clinicians, staff, and patients – have unfavorable views [33]. Known trust issues, algorithmic biases, lack of transparency, and unfairness have deincentivized health systems from adopting AI because it is viewed as an unreliable technology [32,33,37]. Further, health care providers often feel threatened by AI, worried that it will replace their positions. Concern for having AI handle the large, complex tasks of care, they will only perform simple tasks and lose skill over time or have to continuously learn about emerging technologies. Past successes of using AI in health care, however, indicate that it can augment, not replace, care practices. By reconsidering AI as an enabler, health care practices have seen improvements in diagnostics, radiology, analyzing data from wearable technologies, EHR monitoring, use of digital assistants, decision support systems, and breakthroughs in drug discovery, care models, streamlining workflow, and minimizing administrative burdens [32].

## Conclusion

Despite these barriers and unexpected challenges, the results of this pilot study emphasize the transformative potential of AI in clinical research. The successful incorporation of AI into clinical workflows can replace the labor-intensive, time-consuming, and often imprecise process of manual data extraction. The model is being trained on clinical notes from a single institution, and since institutions use individualized note templates with templated free text, the NLP model may not be transferrable to other sites. However, future phases of this project can include data imported from diverse clinical sites to refine the model and expand its capability.

NLP, in particular, holds significant promise as a methodological innovation to address the limitations of extracting symptomatic adverse events from medical records. Future use of more lightweight models or integration of a large language model into the health system may further improve research efficiency. The development of a custom workflow that allowed for parallel processing of thousands of clinical notes simultaneously by a relatively small and inexpensive model. By improving research efficiency across health system networks, AI enables the rapid and consistent identification of symptomatic adverse events among patients treated for cancer. Leveraging these large patient cohorts, researchers can better explore the etiology, management, and mitigation of therapy-related toxicities.

Progress in harnessing the potential of AI in clinical research hinges on successful partnerships between clinical and data science researchers. This transparent account of our journey as a newly formed interdisciplinary team integrating AI into oncology research provides a framework, key lessons, and actionable recommendations for clinicians aiming to explore AI applications. Success is contingent on institutional support—both financial and logistical—and the assembly of a team of data and computer scientists with aligned priorities. Regardless of previous research experience, sufficient time must also be allocated to achieve mutual understanding, acquire new skills, build trust, and foster effective working relationships. By sharing our experience, we are hopeful that readers are empowered to take their first steps with greater confidence, mitigate delays we encountered, and chart a more efficient path toward advancing their own AI-driven research endeavors.

## Abbreviations

AI: artificial intelligence
cTAKES: clinical text analysis and knowledge extraction system
CTCAE: Common Terminology Criteria for Adverse Events
DSBU: Data Science and Biostatistics Unit
EHR: electronic health record
EMR: electronic medical record
HIPAA: Health Insurance Portability and Accountability Act
*ICD-10*: *International Classification of Diseases, Tenth Revision*
*ICD-9*: *International Classification of Diseases, Ninth Revision*
IRB: Institutional Review Board
NLP: natural language processing

## References

[1] Shao D, Dai Y, Li N (2022). Artificial intelligence in clinical research of cancers. Brief Bioinformatics.

[2] Levine AB, Schlosser C, Grewal J, Coope R, Jones SJM, Yip S (2019). Rise of the machines: advances in deep learning for cancer diagnosis. Trends Cancer.

[3] Vamathevan J, Clark D, Czodrowski P (2019). Applications of machine learning in drug discovery and development. Nat Rev Drug Discov.

[4] Crossnohere NL, Elsaid M, Paskett J, Bose-Brill S, Bridges JFP (2022). Guidelines for artificial intelligence in medicine: literature review and content analysis of frameworks. J Med Internet Res.

[5] Bawack RE, Fosso Wamba S, Carillo KDA (2021). A framework for understanding artificial intelligence research: insights from practice. JEIM.

[6] Shah P, Kendall F, Khozin S (2019). Artificial intelligence and machine learning in clinical development: a translational perspective. NPJ Digit Med.

[7] Carlotto A, Hogsett VL, Maiorini EM, Razulis JG, Sonis ST (2013). The economic burden of toxicities associated with cancer treatment: review of the literature and analysis of nausea and vomiting, diarrhoea, oral mucositis and fatigue. Pharmacoeconomics.

[8] Hooke MC, Linder LA (2019). Symptoms in children receiving treatment for cancer-part I: fatigue, sleep disturbance, and nausea/vomiting. J Pediatr Oncol Nurs.

[9] Tay N, Laakso EL, Schweitzer D, Endersby R, Vetter I, Starobova H (2022). Chemotherapy-induced peripheral neuropathy in children and adolescent cancer patients. Front Mol Biosci.

[10] Thornton CP, Orgel E (2023). Dose-limiting mucositis: friend or foe?. Support Care Cancer.

[11] Berman R, Davies A, Cooksley T (2020). Supportive care: an indispensable component of modern oncology. Clin Oncol (R Coll Radiol).

[12] Scott EC, Jewell A (2019). Supportive care needs of people with pancreatic cancer: a literature review. Cancer Nursing Practice.

[13] Snaman J, McCarthy S, Wiener L, Wolfe J (2020). Pediatric palliative care in oncology. J Clin Oncol.

[14] Basch E, Reeve BB, Mitchell SA (2014). Development of the National Cancer Institute’s patient-reported outcomes version of the common terminology criteria for adverse events (PRO-CTCAE). J Natl Cancer Inst.

[15] Trotti A, Colevas AD, Setser A, Basch E (2007). Patient-reported outcomes and the evolution of adverse event reporting in oncology. J Clin Oncol.

[16] Merrow M, King N (2022). Optimizing antiemetic therapy for children undergoing chemotherapy. J Pediatr Nurs.

[17] Smith EML, Kuisell C, Cho Y (2021). Characteristics and patterns of pediatric chemotherapy-induced peripheral neuropathy: a systematic review. Cancer Treat Res Commun.

[18] Miller TP, Li Y, Kavcic M (2016). Accuracy of adverse event ascertainment in clinical trials for pediatric acute myeloid leukemia. J Clin Oncol.

[19] Miller TP, Marx MZ, Henchen C (2022). Challenges and barriers to adverse event reporting in clinical trials: a children’s oncology group report. J Patient Saf.

[20] Hong JC, Fairchild AT, Tanksley JP, Palta M, Tenenbaum JD (2020). Natural language processing for abstraction of cancer treatment toxicities: accuracy versus human experts. JAMIA Open.

[21] Li A, da Costa WL, Guffey D (2022). Developing and optimizing a computable phenotype for incident venous thromboembolism in a longitudinal cohort of patients with cancer. Res Pract Thromb Haemost.

[22] Mashima Y, Tamura T, Kunikata J (2022). Using natural language processing techniques to detect adverse events from progress notes due to chemotherapy. Cancer Inform.

[23] Muñoz AJ, Souto JC, Lecumberri R (2023). Development of a predictive model of venous thromboembolism recurrence in anticoagulated cancer patients using machine learning. Thromb Res.

[24] Zitu MM, Zhang S, Owen DH, Chiang C, Li L (2023). Generalizability of machine learning methods in detecting adverse drug events from clinical narratives in electronic medical records. Front Pharmacol.

[25] Flood EL, Schweig L, Froh EB (2024). The Arcus experience: bridging the data science gap for nurse researchers. Nurs Res.

[26] El-khalek HA, Aziz RF, Morgan ES (2019). Identification of construction subcontractor prequalification evaluation criteria and their impact on project success. Alexandria Engineering Journal.

[27] Gupta K, Walton R, Kataria SP (2021). Chemotherapy-induced nausea and vomiting: pathogenesis, recommendations, and new trends. Cancer Treat Res Commun.

[28] (2017). Common terminology criteria for adverse events v50. National Cancer Institute Cancer Therapy Evaluation Program.

[29] Savova GK, Masanz JJ, Ogren PV (2010). Mayo clinical Text Analysis and Knowledge Extraction System (cTAKES): architecture, component evaluation and applications. J Am Med Inform Assoc.

[30] Thayer J, Pennington JW Fault-tolerant, distributed, and scalable natural language processing with ctakes.

[31] Daniali M, Galer PD, Lewis-Smith D (2023). Enriching representation learning using 53 million patient notes through human phenotype ontology embedding. Artif Intell Med.

[32] Esmaeilzadeh P (2024). Challenges and strategies for wide-scale artificial intelligence (AI) deployment in healthcare practices: a perspective for healthcare organizations. Artif Intell Med.

[33] Kulkov I (2021). The role of artificial intelligence in business transformation: a case of pharmaceutical companies. Technol Soc.

[34] Shaw J, Rudzicz F, Jamieson T, Goldfarb A (2019). Artificial intelligence and the implementation challenge. J Med Internet Res.

[35] Martínez-García M, Hernández-Lemus E (2021). Data integration challenges for machine learning in precision medicine. Front Med (Lausanne).

[36] Sinha S, Lee YM (2024). Challenges with developing and deploying AI models and applications in industrial systems. Discov Artif Intell.

[37] Wubineh BZ, Deriba FG, Woldeyohannis MM (2024). Exploring the opportunities and challenges of implementing artificial intelligence in healthcare: a systematic literature review. Urol Oncol.

